# Effect of plane of nutrition during the first 12 weeks of life on growth, metabolic and reproductive hormone concentrations, and testicular relative mRNA abundance in preweaned Holstein Friesian bull calves

**DOI:** 10.1093/jas/skab195

**Published:** 2021-06-27

**Authors:** Stephen P Coen, Kate Keogh, Colin J Byrne, Pat Lonergan, Sean Fair, Mark A Crowe, David A Kenny

**Affiliations:** 1 Teagasc Animal and Grassland Research and Innovation Centre, Grange, Dunsany, County Meath, Ireland; 2 School of Agriculture and Food Science, University College Dublin, Belfield, Dublin 4, Ireland; 3 Laboratory of Animal Reproduction, Department of Biological Sciences, Biomaterials Research Cluster, Bernal Institute, Faculty of Science and Engineering, University of Limerick, Limerick, Ireland; 4 School of Veterinary Medicine, University College Dublin, Belfield, Dublin 4, Ireland

**Keywords:** early-life nutrition, gene expression, metabolic signaling, neuroendocrine, sexual development

## Abstract

The objective of this study was to examine the effect of nutrition during the first 12 wk of life on aspects of the physiological and transcriptional regulation of testicular and overall sexual development in the bull calf. Holstein Friesian bull calves with a mean (SD) age and bodyweight of 17.5 (2.85) d and 48.8 (5.30) kg, respectively, were assigned to either a high (HI; *n* = 15) or moderate (MOD; *n* = 15) plane of nutrition and were individually fed milk replacer and concentrate to achieve overall target growth rates of at least 1.0 and 0.5 kg/d, respectively. Throughout the trial, animal growth performance, feed intake, and systemic concentrations of metabolites, metabolic hormones, and reproductive hormones were assessed. Additionally, pulsatility of reproductive hormones (luteinizing hormone, follicle-stimulating hormone, and testosterone) was recorded at 15-min intervals during a 10-h period at 10 wk of age. At 87 ± 2.14 d of age, all calves were euthanized, testes were weighed, and testicular tissue was harvested. Differential expression of messenger ribonucleic acid (mRNA) candidate genes involved in testicular development was examined using quantitative polymerase chain reaction assays. All data were analyzed using the MIXED procedure in Statistical Analysis Software using terms for treatment as well as time for repeated measures. Blood metabolites and metabolic hormones generally reflected the improved metabolic status of the calves on the HI plane of nutrition though the concentrations of reproductive hormones were not affected by diet. Calves on the HI diet had greater mean (SED) slaughter weight (112.4 vs. 87.70 [2.98] kg; *P* < 0.0001) and testicular tissue weight (29.2 vs. 20.1 [2.21] g; *P* = 0.0003) than those on the MOD diet. Relative mRNA abundance data indicated advanced testicular development through upregulation of genes involved in cellular metabolism (*SIRT1*; *P* = 0.0282), cholesterol biosynthesis (*EBP*; *P* = 0.007), testicular function (*INSL3*; *P* = 0.0077), and Sertoli cell development (*CLDN11*; *P* = 0.0054) in HI compared with MOD calves. In conclusion, results demonstrate that offering dairy-bred male calves a high plane of nutrition during the first 3 mo of life not only improves growth performance and metabolic status but also advances testicular development consistent with more precocious sexual maturation.

## Introduction

Genomically assisted selection has facilitated the reliable identification of young genetically elite bulls within weeks of birth for potential use in artificial insemination (AI) programs (Taylor et al., 2018). However, inherent variability in the timing of sexual maturation can lead to a delay in the availability of semen of sufficient quality for cryopreservation and/or subsequent use in the field (Byrne et al., 2018). This typically results in an imbalance between supply and demand of semen for use in AI which is further accentuated within the context of seasonal dairy production systems, characterized by relatively short breeding seasons (Fair and Lonergan, 2018). Hastening the onset of puberty and subsequent sexual maturation would therefore make semen available from genetically elite sires at a younger age, allowing the industry earlier access to the best genetics, thus expediting genetic progress (Byrne et al., 2018).

Enhanced nutrition during the first 6 mo of life has been clearly shown to accelerate sexual development in bull calves. Indeed, our group (Byrne et al., 2017b) and others (Kenny et al., 2018) have demonstrated that this accelerated development is mediated through complex biochemical interplay between metabolic cues and neuroendocrine signaling within the hypothalamic–pituitary–testicular (HPT) axis, leading to precocious testicular development, steroidogenesis, and spermatogenesis (Kenny et al., 2018). Bull calves typically experience a transient rise in anterior pituitary-derived systemic luteinizing hormone (LH) from approximately 8 to 20 wk of age (Evans et al., 1996). Indeed, calves offered a high plane of nutrition during the first 6 mo of life display earlier and enhanced LH secretion (Thundathil et al., 2016). Enhanced nutrition during this critical early-life period can directly affect hypothalamic gonadotropin-releasing hormone pulsatility, ultimately leading to enhanced LH pulsatility as well as subsequent testosterone (TT) synthesis and release (Brito et al., 2007a; Byrne et al., 2018). This in turn advances testicular development and steroidogenesis, ultimately leading to earlier onset of puberty and sexual maturation (Brito et al., 2007a; Dance et al., 2015). Moreover, it is also evident that delayed onset of puberty as a consequence of undernutrition during early calfhood cannot be circumvented thereafter through subsequent dietary augmentation (Brito et al., 2007a; Byrne et al., 2018), highlighting the important latent effect of early-life management for subsequent reproductive development. However, in a study carried out on Holstein Friesian bull calves, it was found that bull calves fed a high-energy diet from 8 to 33 wk of age experienced advanced aspects of sexual maturation and increased testes size, but hastened puberty or sperm production was not reflected (Harstine et al., 2015).

While the positive impact of early-life nutrition on sexual development in the bull is clear, the optimum window of development to target, as well as the precise underlying molecular mechanisms involved, are yet to be fully elucidated (Kenny et al., 2018). Our group has previously reported that a high-energy plane of nutrition (high-energy diet vs. low-energy diet) during the first 18 wk of life altered the physiological and molecular control of the hypothalamus (arcuate nucleus region), anterior pituitary, and testes in the bull calf (English et al., 2018a), consistent with advancing puberty by approximately 4 wk (Byrne et al., 2017a). While our previous molecular-based evaluations have provided insight into the impact of early-life plane of nutrition on HPT regulation, it is not clear whether these differences observed at 18 wk of age were a result of biochemical events that occurred earlier during the aforementioned prepubertal LH transient rise which typically occurs between 8 and 20 wk of age (Rawlings and Evans, 1995; Evans et al., 1996) or prior to this time. Consequently, in order to more precisely study the effect of metabolic status on aspects of the physiological and molecular regulation of reproductive and metabolic development, the current study was focused on an earlier period of development (up to 12 wk of age) which coincides with the start of the prepubertal gonadotrophin rise. We hypothesized that offering calves a high plane of nutrition during early calfhood (2 to 12 wk of age) affect the endocrinological and transcriptional control of the HPT, consistent with accelerated testicular development and precocious onset of puberty.

## Material and Methods

This study was conducted at the Teagasc Animal and Grassland Research and Innovation Centre. All procedures involving animals were approved by the Teagasc Animal Ethics Committee and licensed by the Irish Health Products Regulatory Authority in accordance with the European Union Directive 2010/63/EU.

### Experimental design and animal management

In total, 30 Holstein Friesian bull calves were used for this study. All calves were sourced from four commercial dairy herds in Ireland, and following an acclimatization period (5 to 7 d) (English et al., 2018b), all calves were blocked on age, sire, initial body weight, and farm of origin and were randomly assigned to one of two dietary groups: a high (HI, *n* = 15) or a moderate (MOD, *n* = 15) plane of nutrition. The 30 calves used in this study were derived from 17 separate sires with a maximum of 3 calves bred by any one sire. Mean (SD) age and liveweight of calves were 17.5 (2.85) d and 48.8 (5.3) kg, respectively. Throughout the 10-wk dietary trial period, all calves were individually fed milk replacer (MR; Heiferlac, Volac International, Orwell, Royston, Hertfordshire; recommended feeding rate of 900 g/d; Table 1) and pelleted concentrate (Table 1) using an electronic feeding system (Forster-Tecknik Vario, Engen, Germany). Calves in the HI group received 1,500 g of MR reconstituted offered at a rate of 10 L/d with ad libitum concentrate, while MOD calves received 500 g of MR reconstituted offered at a rate of 4 L/d plus a maximum of 0.5 kg of concentrate daily. The MOD plane of nutrition has a MR reconstitution rate of 12.5%, reflecting common commercial practice for male dairy-bred calves. The HI diet has a reconstitution rate of 15% reflecting the higher end of the scale to support high-performance rearing programs. Additionally, all calves had ad libitum access to fresh water and approximately 0.5 kg of hay daily. Dietary regimes for each group were designed to achieve overall target growth rates of at least 1.0 or 0.5 kg/d for HI and MOD calves, respectively. Liveweight was recorded on a weekly basis using an electronic scale. Feed intake and feeding behavior characteristics were measured via the electronic feeding system including all visits with or without feed entitlement.

**Table 1.** Chemical composition of concentrate and MR fed to Holstein Friesian bull calves from 2 to 12 wk of age

Composition	Concentrate	MR
Dry matter, g/kg	906	960
Crude protein, g/kg DM^1^	209.77	281.72
Ash, g/kg DM	90.43	–
Crude oil^2^, g/kg DM	49.60	32.73

^1^DM, dry matter.

^2^Concentrate measured using ether extraction, and MR measured using acid hydrolysis.

### Blood sample collection

Upon arrival, a blood sample was collected from each calf through jugular venepuncture into a 9 mL BD Serum Separator Tube II Advance tubes (BD Vacutainer, Unitech, Dublin, Ireland). The serum was harvested and centrifuged at 1,600 × *g* for 10 min at 4 °C, and the samples were stored at −20 °C pending zinc sulfate turbidity (ZST) analysis. The ZST test (proxy for immunoglobulin status; Earley et al., 2000) was performed on serum samples with the turbidity subsequently measured at 520 nm using a spectrophotometer (McEwan et al., 1970). Blood samples were collected from all calves through jugular venepuncture as previously described by Byrne et al. (2018) at the beginning of the trial (baseline, 2 wk of age), midpoint (7 wk of age), and prior to slaughter (12 wk of age). All calves were blood sampled approximately 1 h after morning feeding (08:00 a.m.). Blood samples were assessed for circulating concentrations of metabolites (beta hydroxybutyrate [BHB], glucose (non-fasting), non-esterified fatty acids [NEFAs], and cholesterol), metabolic hormones (insulin-like growth factor-1 [IGF-1] and insulin), and reproductive hormones (anti- Müllerian hormone [AMH], LH, follicle-stimulating hormone [FSH], and TT).

At 10 wk of age, an intensive, non-stimulated, 10-h window blood sampling regime was undertaken on all calves to determine diurnal variation in FSH, LH, and TT. To facilitate frequent blood collection, all calves were aseptically fitted with an indwelling jugular catheter 12 h prior to the commencement of the intensive blood sampling regime. Blood samples were collected from all calves at 15-min intervals over 10 h for the evaluation of circulating concentrations of gonadotropins (LH and FSH; *n* = 40 timepoints per calf). Additionally, blood samples were also harvested at 60-min intervals for determination of TT concentrations. Blood samples were processed as previously described (Byrne et al., 2018). Blood samples were collected into 9-mL evacuated tubes containing lithium heparin (Greiner Vacuette, Cruinn Diagnostics, Dublin, Ireland) and subsequently analyzed for IGF-1, BHB, and NEFAs. For insulin analysis, blood was collected into a 6-mL K3-EDTA (Vacuette, Cruinn Diagnostics) tube. For glucose analysis, blood was collected into a 4-mL sodium fluoride (Greiner Vacuette, Cruinn Diagnostics) tube. Blood samples were centrifuged at 1,750 × *g* for 15 min, and plasma was collected and stored at −20 °C before analysis. Blood samples were also collected into a 9-mL evacuated serum separator tube (Becton Dickinson, Dublin, Ireland) at the same time points. Blood in serum separator tubes was subsequently allowed to clot overnight and then centrifuged at 800 × *g* for 10 min; serum was harvested and stored at −20 °C pending analysis, outlined below.

### Tissue sampling

At 12 wk of age (mean (SD) age: 87 [2.14] d), all calves were euthanized through intravenous administration of an overdose of sodium pentobarbitone (300 mg/mL: 0.25 mL/kg bodyweight). Death was determined through the absence of ocular response. Following euthanasia, the testes were excised from each calf. Testes were dissected to remove the epididymides and tunica albuginea as well as any excess connective tissue and paired testes weight was recorded. A section of the parenchyma was dissected from the middle of one testis from each calf, washed in sterile Dulbecco’s phosphate-buffered saline, and subsequently snap frozen in liquid nitrogen. Samples were then stored at −80 °C pending further processing.

### Metabolite assays

Metabolite concentrations (BHB, NEFAs, glucose, and cholesterol) were determined using commercial biochemical assay kits (Olympus Diagnostics, Tokyo, Japan, and Randox Laboratories LTD, Co. Antrim, Northern Ireland), with all assays conducted on a Beckmann Coulter AU 400 clinical analyzer (Olympus Diagnostics). Coefficient of variation (CV) percentages for a standard for each metabolite assay were as follows: 5.37% for glucose, 1.56% for BHB, 5.5% for cholesterol, and 1.55% for NEFAs.

### Metabolic hormones

Concentrations of the metabolic hormones, insulin and IGF-1, were determined through immunoradiometric assay (IRMA) and radioimmunoassay (RIA), respectively. Specifically, insulin concentrations were determined through IRMA as previously described by Ochocińska et al. (2016) (DIAsource Immunoassays SA, Louvain-la-Neuve, Belgium). Insulin assay sensitivity was 4.56 ng/mL, while intra-assay CVs for low, medium, and high standards were 10.13%, 9.79%, and 8.11%, respectively. Plasma concentrations of IGF-1 were determined through RIA, which included an acid-ethanol extraction and Tris neutralization procedure of plasma samples prior to RIA as previously described (Beltman et al., 2010). Inter-assay CVs for low, medium, and high IGF-1 samples were 7.40%, 3.33%, and 3.08%, respectively, with intra-assay CVs of 1.72%, 5.60%, and 1.29% reported for low, medium, and high IGF-1 samples, respectively. Overall, the sensitivity of the IGF-1 assay was 3.90 ng/mL.

### Reproductive hormones

Serum samples were utilized for the evaluation of FSH, LH, and TT through RIA and AMH through enzyme-linked immunosorbent assay (ELISA). Concentrations of FSH were determined as previously described by Crowe et al. (1997). The sensitivity of the FSH assay was 0.025 ng/mL and the mean inter-assay CVs (*n* = 6) for serum samples containing 1.4, 1.6, and 3.6 ng/mL were 1.0%, 2.9%, and 3.8%, respectively, while the intra-assay CVs (*n* = 8) for the same samples were 9.2%, 8.1%, and 10.4%, respectively. Serum LH was quantified using the method of Cooke et al. (1997) with the following modifications: aliquots of serum or standard (200 µL; USDA-bLH-1-2 [AFP11118B] NHPP, Bethesda, MD), monoclonal antibody (150 µL; 518B7 anti-LH-B; 1:150,000 dilution; supplied by J. Roser, University of California, Davis, CA), βLH (100 µL; USDA bLH-I-1 (AFP-11743B) NHPP, Bethesda, MD), and I125 radio-ligand (approximately 12,000 c.p.m. per tube) were added to 12- × 75-mm polystyrene tubes and vortex-mixed and incubated at room temperature for 24 h. Following this, 1% Normal Mouse serum (100 µL; Invitrogen 10410) and 1% Goat Anti-Mouse (1 mL; GAMG80-0100 Equitech-Bio, Inc.) in 5% poly-ethylene glycol were added to each tube, vortexed, and then incubated for 1 h at room temperature. Following this, tubes were centrifuged for 20 min at 448 × *g*. Resultant supernatant was poured off and radioactivity of iodinated LH in the retained precipitate was determined using a gamma counter. The sensitivity of the LH RIA assay was 0.063 ng/mL. Inter- and intra-assay CVs for LH were 5.27%, 5.42%, and 4.49%, and 6.58%, 3.13%, and 6.11% for low-, medium-, and high-LH quality controls, respectively. The Testo-RIA-CT DIAsource Immunoassay was employed for the quantification of TT in serum samples by following the manufacturer’s instructions. The assay’s sensitivity was 0.05 ng/mL. Inter- and intra-assay CVs for TT were 10.50% and 5.77%, and 8.76% and 7.88% for medium- and high-TT quality controls, respectively. Concentrations of AMH were determined through a commercial bovine AMH ELISA kit (Ansh Labs, TX), according to the manufacturers’ instructions. The sensitivity of the AMH assay was 0.011 ng/mL and the mean intra-assay CVs were 5.34%, 3.53%, and 9.62% for low, medium, and high standards, respectively.

### Relative mRNA abundance

Using approximately 50 mg of testes parenchyma as starting material, total RNA was isolated from all samples using the RNeasy Universal plus Kit (Qiagen, Manchester, UK) according to the manufacturer’s instructions. Yield of resultant RNA for each sample was assessed by measuring the absorbance at 260 nm using a Nanodrop spectrophotometer ND-1000 (Nanodrop Technologies, Wilmington, Germany). RNA quality was then evaluated using the RNA 6000 Nano Lab Chip kit (Agilent Technologies, Cork, Ireland) on an Agilent Bioanalyzer 2100. All RNA samples had a RNA integrity number of at least 7, with values ranging between 7.8 and 9.8 across all samples. Using 2 μg of high-quality RNA, the High-Capacity complementary DNA (cDNA) Reverse Transcription Kit (Applied Biosystems, Foster City, CA) in combination with Multiscribe reverse transcriptase was employed to reverse transcribe total RNA into cDNA, following the manufacturer’s instructions. cDNA samples were then stored at −20 °C pending further processing.

The Primer3 software (http://primer3.ut.ee/) was employed to design primers for both target genes of interest and reference genes (Koressaar and Remm, 2007). Resultant primer sequences from Primer3 were checked for gene specificity using the BLAST tool within NCBI (http://www.ncbi.nlm.nih.gov/BLAST/). The genes examined in this study along with their primer sequences are outlined in Table 2. All primers used were obtained commercially (Sigma-Aldrich Ireland, Dublin, Ireland).

**Table 2.** Gene ID, sequence, and accession number for all genes assessed in the testes of 12-wk-old Holstein Friesian bull calves offered a high (HI) or moderate (MOD) plane of nutrition from 2 to 12 wk of life

Gene^1^	Primer sequence (5′–3′)	Accession number
*FSHR*	FWD: CGCTGGAAAGATGGCATACC RVS: GCTCACCTTCATGTAGCTGC	NM_174061.1
*LHR*	FWD: GGGAAATCAGCGTTGTCCCATTGA RVS: GCATCCACAAGCTTCCAGATGTTACGA	NM_174381.1
*AR*	FWD: AGCCTCAATGAACTGGGTGA RVS: AACACCATAAGCCCCATCCA	NM_001244127.1
*IGF1R*	FWD: TTTCTCAATGAGGCCTCGGT RVS: CTGCAATCTCACCAGCCATC	NM_001244612.1
*PCNA*	FWD: GGCGTTCATAGTCGTGTTCC RVS: CTTCAAGATGGAGCCCTGGA	NM_001034494.1
*StAR*	FWD: GCCAGGAAAGATGCTTCTCG RVS: GACGCCGAACCTGGTTAATC	NM_174189.3
*INSL3*	FWD: GTGAACTCCTGATGCCACAC RVS: TTTGGGGTCTGGTGGTATCC	NM_174365.2
*CLDN11*	FWD: GCCTCTCTGTTCCCTCTCTC RVS: CCCACCCCACATTCCCTATT	NM_001035055.2
*THY1*	FWD: CCTCACCTCTGCCAATACCA RVS: ACGGAAGCAGCTCTGGAATA	NM_001034765.1
*UCHL1*	FWD: GCTTCTCTGGGTTGTGTTCG RVS: GGTCAGCACTTTGTTCAGCA	NM_001046172.2
*IGF-1*	FWD: ATGCCCAAGGCTCAGAAG RVS: GGTGGCATGTCATTCTTCACT	NM_001077828
*GHR*	FWD: ATGGCGGTATTGTGGATCAT RVS: AGGATGTCGGCATGAATCTC	NM_176608.1
*FOS*	FWD: TTTGACTGCTCGCGATCATG RVS: CAGATCGGTGCAGTAGTCCT	NM_182786
*SIRT1*	FWD: CCAACGGTTTCCATTCGTGT RVS: TCGAGGATCTGTGCCAATCA	NM_001192980.1
*PIK3*	FWD: AAATGCGGCGACCAGATTTC RVS: AAGCCTGAGATTTCCCAGCT	NM_174574.1
*EIF4E*	FWD: AATGCCTGGCTGTGACTACT RVS: ATTAGCCATCGTCCTCCTCG	NM_174310.3
*MTOR*	FWD: CACAAGTCGAGCTGCTCATC RVS: CTGTGCCTCCAGTTACCAGA	XM_015466779.1
*EIFEBP1*	FWD: CCCCTTCTTCTTCGTGGACA RVS: CTCTTCTGTGTCCACTTGCC	NM_001077893.2
*PRKAA1*	FWD: TGTCACAGGCATATGGTGGT RVS: GTGCAGCATAGTTGGGTGAG	NM_001109802.2
*INSR*	FWD: TGAAGCCAAGGCAGATGATATT RVS: GCCACATCAAGTGAACAACGTT	XM_590552.4
*AKT*	FWD: ACATCCGAGACCAGTCCAAG RVS: TGTCGAGTGTGTCTGCGTAT	NM_001191309
*EBP*	FWD: TGGTACCCGGACTCCTCATA RVS: AGCCGGTGTTTAAGCCTAGT	NM_001034500.1
*GAPDH*	FWD: AGATGGTGAAGGTCGGAGTG RVS: TGACTGTGCCGTTGAACTTG	NM_001034034.2
*UBQ*	FWD: CACGTTTGGGAGTTGTGCTT RVS: TCTCTTCCACCATTCCCACC	NM_001206307
*YWHAZ*	FWD: GATCCCCAACGCTTCACAAG RVS: TTGTCATCACCAGCTGCAAC	NM_174814.2
*RPS9*	FWD: GCTGACGCTGGATGAGAAAG RVS: ATCCAGCTTCATCTTGCCCT	NM_001101152.2

^1^*AKT*, serine/threonine kinase 1; *AMH*, anti-Müllerian hormone; *AR*, androgen receptor; *CLDN11*, claudin 11; *EBP*, cholestenol delta-isomerase; *EIF4E*, eukaryotic translation initiation factor 4E; *EIFEBP1*, eukaryotic translation initiation factor 4E-binding protein 1; *FOS*, Fos proto-oncogene, AP-1 transcription factor subunit; *FSHR,* follicle-stimulating hormone receptor; *GAPDH*, glyceraldehyde-3-phosphate dehydrogenase; *GATA4*, GATA binding protein 4; *GHR*, growth hormone receptor; *IGF-1*, insulin-like growth factor 1; *IGF1-R*, insulin-like growth factor 1 receptor; *INSL3*, insulin-like 3; *INSR*, insulin receptor; *LHR,* luteinising hormone receptor; *Mtor*, mechanistic target of rapamycin kinase; *MVK*, mevalonate kinase; *PCNA*, proliferation cell nuclear antigen; *PIK3*, phosphatidylinositol-4,5-bisphosphate 3-kinase catalytic subunit gamma; *PRKAA1*, protein kinase AMP-activated catalytic subunit alpha 1; *RPS9*, ribosomal protein S9; *StAR*, steroidogenic acute regulatory protein; *THY1*, Thy-1 cell surface antigen; *UBQ*, ubiquitin; *UCHL1*, ubiquitin carboxyl-terminal esterase L1; *YWHAZ*, tyrosine 3-monooxygenase/tryptophan 5-monooxygenase activation protein, zeta.

Relative messenger ribonucleic acid (mRNA) abundance assays were undertaken on an ABI 7500 Fast Real-Time PCR System (Applied Biosystems, Warrington, UK) as per the protocol described by Keogh et al. (2015). In order to determine polymerase chain reaction (PCR) amplification efficiencies, the *E* = 10^(−1/slope) − 1 formula was applied to cycle threshold (Ct) values of pooled serial diluted cDNA samples (Bustin et al., 2009). Primers with PCR efficiencies between 90% and 110% were deemed acceptable. The expression of four reference genes, previously used for bovine testes relative mRNA abundance (English et al., 2018b), was also evaluated across all testes cDNA samples for determination of the most stably expressed reference gene. These included ribosomal protein S9 (*RPS9*), glyceraldehyde-3-phosphate dehydrogenase (*GAPDH*), tyrosine 3-monooxygenase/tryptophan 5-monooxygenase activation protein, zeta (*YWHAZ*), and ubiquitin (*UBQ*). The stability of the resultant Ct values for each reference gene tested was then determined using GeNorm software (GenEx 5.2.1.3; MultiD Analyses, Gothenburg, Sweden) by calculating both the intra- and intergroup CV. GeNorm analysis provides a stability value (*M* value) (Vandesompele et al., 2002), with a lower *M* value indicating greater relative mRNA abundance stability across all samples tested. Of the four reference genes evaluated, *YWHAZ* and *RSP9* both had the lowest *M* value (0.93; *GAPDH* = 1.156; *UBQ* = 1.02) and thus were selected as suitably stable reference genes. Following completion of quantitative PCR, resultant Ct values were analyzed using GenEx software (www.multid.se/genex.html). All amplified PCR products were sequenced to verify their identity (Macrogen Europe, Amsterdam, the Netherlands) and all amplicons were confirmed 100% homologous to their target sequence.

### Statistical analysis

In order to determine the magnitude of response of reproductive hormone profiles (FSH, LH, TT) from the 10-h window bleed, these data were analyzed using area under the curve (AUC) analysis using Sigma Plot, version 14.0 (Systat Software, San Jose, CA). All data were analyzed using the procedures of Statistical Analysis Software (SAS version 9.4). For this, all data were first tested for normality of distribution using the UNIVARIATE procedure and, where appropriate, were transformed to the power of lambda using the TRANSREG procedure. Differences between treatment groups were compared using the MIXED procedure in SAS. For all parameters assessed, block was included as a random effect with dietary treatment included as a fixed effect within the statistical model. For any repeated measurements, including bodyweight, blood analytes, and feed intake, data were analyzed with terms for dietary treatment and time as well as their interaction. The type of variance–covariance structure for each variable was determined based on the magnitude of the Akaike information criterion (AIC) for models run including compound symmetry, unstructured, and autoregressive covariance structures, with the model with the lowest AIC coefficient used for final analysis. All results are presented as mean ± SEM unless stated otherwise. Mean values were considered to be statistically significantly different when *P* ≤0.05.

## Results

### Animal performance, feed intake, and feeding behavior

ZST test performed on serum collected from all calves upon arrival at Grange research farm showed no difference between calves on HI and MOD dietary treatments (*P* = 0.8635). ZST values ranged between 19 and 41 units across all calves used in the study. Additionally, throughout the duration of the dietary trial, there was no evidence for any health-related issues with any of the calves employed in this study. Animal growth performance and dietary intake results are presented in Table 3. Milk consumption reflected the experimental design with calves on the HI plane of nutrition consuming 2.75 times more MR (kg/dry matter) than those in the MOD group (*P* < 0.0001). Calves offered HI had a greater number of visits rewarded with feed, than calves offered MOD (*P* < 0.0001), with the inverse observed in relation to visits without entitlement (*P* < 0.0001). As per the experimental design of differential feeding, average daily gain was 35% higher for calves on the HI compared to the MOD treatment (*P* < 0.0001). Additionally, at slaughter, HI calves were 24.7 kg heavier (*P* < 0.0001; Table 3), with a consistent effect of plane of nutrition on growth rate observed throughout the trial period (*P* < 0.0001; Table 3). Paired testicular weight at slaughter was also higher for HI compared with MOD calves (*P* = 0.0003; Table 3). However, when analyzed as a proportion of body weight, there was no difference between HI and MOD groups for proportional testes weight (*P* = 0.1530; Table 3).

**Table 3.** Effect of plane of nutrition on performance, feeding behavior, and testes tissue weight of Holstein Friesian bull calves (*n* = 30) offered a high (HI) or moderate (MOD) plane of nutrition from 2 to 12 wk of life

	HI	MOD	SEM	*P*-value
Average daily gain, kg	0.88	0.58	0.019	<0.0001
Slaughter weight, kg	112.40	87.70	2.10	<0.0001
MR intake, kg DM/d^1^	1.35	0.49	0.005	<0.0001
Concentrate intake, kg DM/d	0.52	0.41	0.012	0.0868
Visits with entitlement^2^	5.22	2.21	0.044	<0.0001
Visits without entitlement^3^	5.01	13.31	0.18	<0.0001
Testes, g	29.2	20.10	1.56	0.0003
Testes weight, g/kg BW^4^	2.58 × 10^3^	2.3 × 10^3^	0.000013	0.1530

^1^DM, dry matter.

^2^Visits with entitlement: visits to feeder where feed is dispensed.

^3^Visits without entitlement: visits to feeder where feed is not dispensed.

^4^BW, body weight.

### Metabolic hormones and metabolites

Metabolic hormones and metabolite data are presented in Table 4. There was no treatment-by-week interaction for glucose (*P* = 0.83) or cholesterol (*P* = 0.28) concentrations. However, interactions were apparent for IGF-1 (*P* < 0.0001; Table 4), NEFA (*P* = 0.036; Table 4), and BHB (*P* = 0.0061; Table 4). Systemic concentrations of IGF-1 increased as time progressed but were greater in HI compared to M calves. There was a treatment-by-week interaction for IGF-I concentration with calves on a HI plane of nutrition exhibiting greater (*P* < 0.0001) concentrations of IGF-1 than those on a MOD plane of nutrition from 3 wk of age onwards. NEFA concentrations were also greater in HI calves throughout the trial with concentrations changing depending on timepoint. BHB concentrations were greater in MOD calves throughout the trial, with concentrations increasing with increasing age across all calves. There was no effect of treatment on cholesterol concentrations (*P* = 0.056); however, cholesterol concentrations reduced over time (*P* = 0.0008). Glucose followed a similar pattern to cholesterol with concentrations greater in HI (*P* = 0.003) and reduced with increasing age across all calves (*P* < 0.0001). In response to increased circulating glucose concentrations, systemic insulin was also greater in HI calves (*P* = 0.048); however, an effect of time on insulin concentrations was not observed (Table 4).

**Table 4.** Effect of plane of nutrition and week of sampling on plasma metabolites in Holstein Friesian bull calves (*n* = 30) offered a high (HI) or moderate (MOD) plane of nutrition from 2 to 12 wk of age

	Diet		SEM	Age			SEM	*P*-value		
Blood metabolite	HI	MOD		2 wk	7 wk	12 wk		Treatment	Week	Treatment × week
Cholesterol, mmol/L	2.44	2.22	0.078	2.43	2.43	2.15	0.07	0.056	0.0008	0.28
Glucose, mmol/L	5.98	5.23	0.16	6.04	5.82	4.95	0.16	0.003	<0.0001	0.83
BHB, mmol/L	0.09	0.197	0.006	0.08	0.15	0.20	0.009	<0.0001	<0.0001	0.0061
NEFA, mmol/L	0.10	0.08	0.009	0.09	0.097	0.08	0.008	0.12	0.14	0.036
Insulin, μl/mL	22.88	15.35	3.498	20.01	18.98	15.62	4.36	0.048	0.97	0.16
IGF-1, ng/mL	333.31	153.64	11.63	112.08	278.54	339.81	12.66	<0.0001	<0.0001	<0.0001

### Reproductive hormones

The effect of treatment and week of measurement on reproductive hormone profiles is presented in Table 5. There was no evidence (*P* > 0.05) for a treatment-by-week interaction for any hormones assessed (AMH, TT, FSH, and LH). However, an effect of week of sampling was evident for AMH, TT, and FSH, with all three hormones increasing in concentration as the calves got older (*P* < 0.0001). Additionally, although there was no clear effect of treatment on the majority of the reproductive hormones evaluated, TT concentrations were greater in HI calves (*P* = 0.000636) compared to MOD. The intensive blood sampling regimen conducted at 10 wk of age, however, revealed an effect of dietary treatment on circulating concentrations of FSH (*P* = 0.0219) over a 10-h period based on AUC analysis (Table 6), with concentrations greater in the HI compared to the MOD calves. There was no effect of dietary treatment on LH concentrations based on the window bleed results (*P* = 0.2014). Systemic concentrations of TT, FSH, and LH over the 10-h intensive blood sampling regimen are presented in Table 5. The effect of treatment and time on concentrations of FSH, LH, and TT pertaining to the 10-h intensive blood sampling regimen also yielded no significant treatment-by-time interactions (*P* > 0.05); however, significant treatment effects were apparent for each hormone (LH, *P* = 0.0057; FSH, *P* < 0.0001; TT, *P* = 0.0009), with effects of sampling time also apparent for TT (*P* = 0.0018) and LH (*P* = 0.0015).

**Table 5.** Effect of plane of nutrition and month on reproductive hormone concentrations in Holstein Friesian bull calves (*n* = 30) fed a high (HI) or moderate (MOD) plane of nutrition from 2 to 12 wk of age

	Diet		SEM	Age			SEM	P-value		
Hormone	HI	MOD		2 wk	7 wk	12 wk		Treatment	Week	Treatment × week
AMH, ng/mL	383.7	376.6	20.5	321.8	381.3	437.4	18.0	0.8101	<0.0001	0.8014
TT, ng/mL	0.038	0.016	0.007	0.008	0.033	0.057	0.063	0.000636	<0.0001	0.0811
LH, ng/mL	0.31	0.17	0.072	0.11	0.26	0.35	0.093	0.1848	0.2532	0.5483
FSH, ng/mL	0.45	0.39	0.032	0.28	0.53	0.46	0.034	0.3021	<0.0001	0.7127

**Table 6.** Effect of plane of nutrition on mean serum TT, FSH, and LH concentrations in 10-wk-old Holstein Friesian bull calves during a 10-h intensive blood sampling regimen^1^

Hormone	HI	MOD	SEM	*P*-value
TT	2636.2	1522.7	324.3	0.0563
LH	202.4	156.5	41.6	0.2014
FSH	369.8	285.0	24.6	0.0219

^1^Values are expressed as AUC for 40 timepoints representing samples collected every 15 min for 10 h for LH and FSH. Values are expressed as AUC for 10 timepoints representing samples collected every 60 min for 10 h for TT.

### Relative mRNA abundance

Testicular relative mRNA abundance data are presented in Table 7. Of the 25 genes evaluated, only 4 genes were affected by early-life dietary augmentation. These included genes involved in cellular metabolism (*SIRT1*; *P* = 0.0282), cholesterol biosynthesis (*EBP*: *P* = 0.007), testes development (*INSL3*; *P* = 0.0077), and Sertoli cell development (*CLDN11*; *P* = 0.0054), all of which were upregulated in the HI compared with the MOD group (Table 7). There was no effect of plane of nutrition on the relative expression of the other genes assessed (*P* > 0.05).

**Table 7.** Effect of plane of nutrition on the relative expression of key candidate genes in testicular tissue of Holstein Friesian bull calves at 12 wk of age^1^

Gene^2^	HI	MOD	SEM	*P*-value
Gonadotropin/androgen receptor				
* FSHR*	4.14	4.03	0.3	0.8046
* LHR*	3.32	2.99	0.2	0.2585
* AR*	2.05	1.94	0.15	0.6266
Cellular growth/proliferation				
* PCNA*	1.83	1.77	0.21	0.8298
* IGF-1*	0.9	0.91	0.14	0.9832
* GHR*	3.22	3.18	0.17	0.855
Steroidogenesis/cholesterol biosynthesis				
* EBP*	5.79	5.06	0.17	0.007
* MVK*	2.39	2.35	0.19	0.8685
* StAR*	2.94	2.26	0.29	0.1099
* AMH*	0.41	0.43	3.5	0.7746
* GATA4*	3.03	3.48	0.28	0.2814
Insulin/IGF-1 receptor signaling				
* IGF1-R*	4.81	4.87	0.21	0.8628
* AKT*	2.003	1.76	0.13	0.2115
* FOS*	2.2	2.28	0.24	0.8301
* INSR*	2.46	2.55	0.15	0.7079
* PRKAA1*	2.79	3.1	0.37	0.5744
* Mtor*	2.08	2.01	0.17	0.7845
* EIF4EBP1*	1.39	1.4	0.14	0.9913
* SIRT1*	2.41	1.81	0.26	0.0282
* EIF4E*	2.28	2.31	0.24	0.9534
* PIK3*	1.18	1.39	0.19	0.4675
Spermatogenesis				
* THY1*	3.3	3.15	0.35	0.7595
* INSL3*	4.02	3.45	0.14	0.0077
* UCHL1*	1.98	1.43	0.23	0.0999
* CLDN11*	3.46	1.98	0.34	0.0054

1The results are relative to the average of the reference genes *YWHAZ* and *RSP9*.

^2^*AKT*, serine/threonine kinase 1; *AMH*, anti-Müllerian hormone; *AR*, androgen receptor; *CLDN11*, claudin 11; *EBP*, cholestenol delta-isomerase; *EIF4E*, eukaryotic translation initiation factor 4E; *EIFEBP1*, eukaryotic translation initiation factor 4E-binding protein 1; *FSHR*, follicle-stimulating hormone receptor; *FOS*, Fos proto-oncogene, AP-1 transcription factor subunit; *GATA4*, GATA binding protein 4; *GHR*, growth hormone receptor; *IGF-1*, insulin-like growth factor 1; *IGF1-R*, insulin-like growth factor 1 receptor; *INSL3*, insulin-like 3; *INSR*, insulin receptor; *LHR*, luteinising hormone receptor; *Mtor*, mechanistic target of rapamycin kinase; *MVK*, mevalonate kinase; *PCNA*, proliferation cell nuclear antigen; *PIK3*, phosphatidylinositol-4,5-bisphosphate 3-kinase catalytic subunit gamma; *PRKAA1*, protein kinase AMP-activated catalytic subunit alpha 1; *RPS9*, ribosomal protein S9; *StAR*, steroidogenic acute regulatory protein; *THY1*, Thy-1 cell surface antigen; *UCHL1*, ubiquitin carboxyl-terminal esterase L1; *YWHAZ*, tyrosine 3-monooxygenase/tryptophan 5-monooxygenase activation protein, zeta.

## Discussion

The main findings of this study are that offering a high plane of nutrition from 2 to 12 wk of age increased the performance and metabolic status of the bull calves while also advancing testicular development and sexual maturity. This was manifested through greater testicular steroidogenesis and a greater expression of key genes involved in testicular function, Sertoli cell development, and Leydig cell differentiation. In agreement with previous studies from our group (Byrne et al., 2018; English et al., 2018b), which concentrated on a later stage of calf development, the testes exhibited an allometric growth pattern and were 45% heavier at 12 wk of age in calves offered a high compared with a moderate plane of nutrition. Previous studies have shown that larger testicular size in the bull calf, as a consequence of improved calfhood nutrition, is consistent with advanced morphological ontogenesis in the form of greater seminiferous tubule development as well as greater Sertoli cell number and spermatogenic capacity (English et al., 2018b). This is important as Sertoli cell number does not increase after puberty in bulls, between birth and puberty, there is an approximate 5-fold increase in the number of Sertoli cells but after that there are no seasonal- or age-related increases in the number of adult Sertoli cells (Hochereau-de Reviers et al., 1987).

The design of our study ensured that calves on the HI diet grew at a much faster rate than their counterparts on the MOD plane of nutrition, leading to a higher bodyweight at 12 wk of age. This was consistent with their greater number of daily rewarded and less unrewarded visits to the electronic feeder. Calves on a MOD plane of nutrition had eight extra unrewarded visits per day, compared with those on a HI plane of nutrition, indicating a lack of satiety.

The systemic concentrations of the various metabolites assessed in this study were within the normal range for calves in positive energy balance, were consistent with other recent studies (Byrne et al., 2018; English et al. 2018b), and, in general, reflected the divergence in average metabolic status and nutrient intake generated by the two contrasting planes of nutrition employed. Glucose concentrations generally reflected the prevailing plane of nutrition, particularly the greater lactose and starch intake of the calves fed the HI diet. Plasma concentrations of BHB were affected by treatment and were low for both groups but increased as concentrate consumption increased toward the end of the trial period, coincident with normal rumen development (Khan et al., 2011). No difference in concentrations of NEFA was observed, indicating that both treatment groups were in an anabolic state throughout the trial similar to that observed by Byrne et al. (2018).

IGF-I has been implicated in mediating the effect of diet on the functionality of the HPT axis (Brito et al., 2007a; Byrne et al., 2018). We observed a 3-fold higher higher concentration of IGF-I in calves on the HI compared with the MOD plane of nutrition, in agreement with the findings of English et al. (2018b). Studies have shown that a peripubertal increase in TT concentration was delayed in bulls with lesser serum IGF-I concentrations from low nutrition (Brito et al., 2007b). This suggests that IGF-I plays a role in regulating Leydig cell function. In the same study, the authors found that serum IGF-I concentrations accounted for 72% and 67% of variation in paired-testes volume and scrotal circumference at any given age, suggesting that IGF-I could regulate testicular growth (Brito et al., 2007b). However, despite an effect of dietary treatment on systemic concentrations of both IGF-1 and insulin, we did not observe a role for intracellular signaling of either of these hormones toward testes tissue development, established through relative mRNA abundance results. Our relative mRNA abundance results did, however, identify greater expression of *SIRT1* in calves on the HI diet compared to those on the MOD diet. *SIRT1* belongs to a family of signaling proteins called sirtuins that are involved primarily in metabolic regulation (Ye et al., 2017), for example, *SIRT1* has been shown to contribute to insulin sensitivity in peripheral tissues (Reverchon et al., 2016). However, more recently, it has been hypothesized that certain sirtuin functions including metabolic regulation may be mechanistically linked to steroid hormone biosynthesis (Bayele, 2019). Indeed, in their study using bovine granulosa cells, Reverchon et al. (2016) found that the adipokine visfatin improves basal and IGF-1-induced steroidogenesis and IGF-1 receptor signaling through SIRT1. Thus, although differences in the expression of genes related to IGF-1 signaling were not apparent in the current study, which may have been due to the age at sampling selected, the aforementioned results suggest a potential link between SIRT1 and IGF-1 and warrants further investigation.

There was no effect of diet on systemic concentrations of LH. Serum concentrations of LH increase from 4 to 5 wk of age to an early postnatal peak at 12 to 16 wk of age, with a decline at 25 wk of age (Evans et al., 1996; Rawlings et al., 2008; English et al., 2018b). Similarly, it has been reported that the concentration of LH receptors in testicular parenchyma of Hereford × Charolais bulls is high postnatally but decreases from 13 to 25 wk of age, possibly due to the decline in fetal Leydig and undifferentiated Leydig progenitor cell numbers (Bagu et al., 2006).

Serum samples taken at 2, 7, and 12 wk of age provided no evidence for an effect of plane of nutrition on FSH concentrations, consistent with the work of English et al. (2018b) in bulls at 18 wk of age. The higher concentration of FSH observed in the HI calves during the 10-h intensive bleed conducted at 10 wk of age is consistent with the findings of English et al. (2018b), where a strong tendency towards higher systemic concentrations of FSH was observed in calves on a HI plane of nutrition at 10 wk of age (English et al., 2018b). Work carried out on Angus and Angus × Charolais bulls receiving adequate nutrition reported that FSH concentrations typically peak at between 10 and 14 wk of age in those breed types (Brito, 2014).

AMH controls the regression of Müllerian ducts in the male fetus (Rota et al., 2002) and is secreted from prepubertal Sertoli cells from sexual differentiation until puberty (Vigier et al., 1984). AMH secretion typically peaks at birth in male calves, declining thereafter until puberty, as a consequence of increased testicular TT synthesis and secretion, following activation of the HPT axis (Tan et al., 2005; Hero et al., 2012). However, there is some confusion in the literature as to the pattern of secretion of AMH. While some studies report a decline in AMH from birth, others, including ours, observed an increase up to 12 wk of age. We found that, irrespective of dietary treatment, AMH concentrations increased throughout the current trial period and were not affected by diet. AMH concentrations observed in the current study are consistent with a study carried out by Kitahara et al. (2016), who showed that AMH blood concentrations rose from birth until between 2 and 3 mo of age in Japanese Black bull calves and were negatively correlated with blood TT concentration from 4 to 6 mo of age; however, these authors did not study the effect of prevailing plane of nutrition on systemic concentrations of AMH. This would suggest that AMH typically declines as TT synthesis and secretion increases. This also coincides with the differentiation of basal indifferent supporting cells to Sertoli cells, as this starts at 20 wk, and the formation of Sertoli cells completes at approximately 28 wk (Curtis and Amann, 1981). The same study found that gonocytes predominated at 12 wk and by 20 wk most had been replaced by prespermatogonia and A-spermatogonia, suggesting that the transformation from a prepubertal testis to a pubertal testis containing Sertoli cells and A-spermatogonia occurred between 16 and 24 wk (Curtis and Amann, 1981).

We observed a difference in systemic concentrations of TT between calves offered the two nutritional regimens. Previous authors have shown that a higher plane of nutrition has a positive influence on systemic concentrations of TT (English et al., 2018b). Indeed, early elevation in testicular TT secretion in the bull calf is consistent with earlier onset of puberty (Brito et al., 2007a; Byrne et al., 2018). Blood concentrations of TT have been shown to increase in beef bred bull calves following the early LH rise, which typically occurs between 10 and 18 wk of age (Amann, 1983; Rawlings and Evans, 1995). The greater TT concentration in calves on the high plane of nutrition is consistent with the higher systemic concentrations of IGF-I in the calves on that treatment. This is also consistent with cholesterol concentrations, as although not significantly different between treatment groups in our study, there was a tendency for cholesterol to be greater in the HI calves. Cholesterol is the building block of TT where Leydig cells derive most of what they need to produce TT by absorbing cholesterol in the blood. With cholesterol playing a role as a precursor for TT, studies have shown that the higher the cholesterol content, the higher the TT concentration in bulls (Amerkhanov et al., 2014). Moreover, evidence for alterations in cholesterol biosynthesis between the two dietary treatment groups was established through the differential expression of *EBP*. The EBP protein catalyzes the conversion of delta (8)-sterols to their corresponding delta (7)-isomers, playing a major role in cholesterol biosynthesis. Testicular expression of *EBP* was greater in the HI compared to the MOD group in the current study and is consistent with their higher systemic concentrations of TT and in agreement with previous findings from our group (English et al., 2018b).

Despite dietary treatment only affecting TT through the regular blood sampling regime and FSH through the intensive 10-h bleed, we did identify evidence for dietary treatment effects related to the expression of genes involved in spermatogenesis. This was manifested through the greater expression of *CLDN11* and *INSL3* in the HI dietary treatment group. *CLDN11* encodes an obligatory protein involved in tight junction formation and barrier integrity in the testis (Mazaud-Guittot et al., 2010). It is expressed by Sertoli cells, and spermatogenesis does not proceed beyond meiosis in its absence, resulting in male sterility (Mazaud-Guittot et al., 2010). Indeed, the relative abundance results of the *CLDN11* gene of the current study are consistent with the findings of a previous study conducted at our laboratory by English et al. (2018b) where it was shown that offering bull calves a HI compared with a MOD plane of nutrition increased expression of *CLDN11* with transcript abundance in the high group similar to that of mature bulls (used as positive controls) in that study. Additionally, in our study, calves that experienced a HI plane of nutrition in early life displayed higher testicular expression of *INSL3* compared with their contemporaries on the MOD diet. Insulin-like 3 (INSL3) is a major secretory product of testicular Leydig cells (Hannan et al., 2015) and serves as an excellent marker for Leydig cell differentiation and functional capacity during sexual development of cattle (Ivell et al., 2014). In agreement, another recent study from our group showed that a high plane of nutrition in the first 6 mo of life, but not later, increased systemic concentrations of *INSL3* in young bulls (Anand-Ivell et al., 2019). Moreover, in that study, *INSL3* concentration at 4 mo of age correlated well (negatively) with the timing of puberty, as well as with testis size at 18 mo (Anand-Ivell et al., 2019). In agreement, Sakase et al. (2018) found that blood *INSL3* concentrations may be a robust and repeatable biomarker for determining total testicular volume and degree of sexual maturity in prepubertal bull calves. Thus, our results indicate greater testes development and spermatogenesis potential in calves at 12 wk of age offered an enhanced dietary regimen.

## Conclusion

This study provides clear evidence that offering a high plane of nutrition, resulting in a much-improved metabolic status, accelerated the molecular and physiological ontogeny of development of the testes in young bull calves during the first 12 wk of life. This was manifested in the form of increased testicular tissue growth and TT synthesis and secretion as well as upregulation of the expression of key genes involved in testicular function viz. Sertoli cell development and Leydig cell differentiation. The period under investigation in the current study is consistent with the window of development identified at 8 to 20 wk of age, as being central to the early activation of the HPT axis, thus leading to more precocious onset of puberty and sexual maturation. Results are consistent with our earlier published reports which were focused on a later stage of calfhood development and provide new insight and further evidence for the central role of early-life nutritional status in regulating the rate of sexual development and, in particular, testicular cellular functionality in the bull calf. In the current study, we have identified that these positive effects are manifested as early as 12 wk of age, during the typical preweaning phase, thus highlighting the central role of postnatal nutritional management in shortening the generation interval for genetically elite cattle.

## Abbreviations

AI: artificial insemination
AIC: Akaike information criterion
AMH: anti-Müllerian hormone
AUC: area under the curve
BHB: beta hydroxybutyrate
cDNA: complementary DNA
ELISA: enzyme-linked immunosorbent assay
FSH: follicle-stimulating hormone
HPT: hypothalamic–pituitary–testicular
IGF1: insulin-like growth factor 1
IRMA: immunoradiometric assay
LH: luteinizing hormone
MR: milk replacer
mRNA: messenger ribonucleic acid
NEFA: non-esterified fatty acid
PCR: polymerase chain reaction
RIA: radioimmunoassay
TT: testosterone
ZST: zinc sulfate turbidity
